# Distal renal tubular acidosis without renal impairment after use of tenofovir: a case report

**DOI:** 10.1186/s40360-016-0100-y

**Published:** 2016-11-21

**Authors:** Kentaro Iwata, Manabu Nagata, Shuhei Watanabe, Shinichi Nishi

**Affiliations:** 1 Division of Infectious Diseases Therapeutics, Kobe University Graduate School of Medicine, Kusunokicho 7-5-2, Chuoku, Kobe, Hyogo Japan 650-0017; 2 Division of Nephrology and Kidney Center, Kobe University Graduate School of Medicine, Kusunokicho 7-5-2, Chuoku, Kobe, Hyogo Japan 650-0017

**Keywords:** HIV, Renal tubular acidosis, Tenofovir, Case report

## Abstract

**Background:**

Tenofovir, one of antiretroviral medication to treat human immunodeficiency virus (HIV) infection, is known to cause proximal renal tubular acidosis such as Fanconi syndrome, but cases of distal renal tubular acidosis had never been reported.

**Case presentation:**

A 20-year-old man with HIV infection developed nausea and vomiting without diarrhea after starting antiretroviral therapy. Arterial blood gas revealed non-anion-gap metabolic acidosis and urine test showed positive urine anion gap. Tenofovir, one of antiretroviral medicine the patient received, was considered to be the cause of this acidosis and all antiretroviral drugs were discontinued. Symptoms disappeared promptly without recurrence of symptoms after resuming antiretroviral medications without tenofovir.

**Conclusion:**

Distal renal tubular acidosis caused by tenofovir, without renal impairment is very rare. Since causes of nausea and vomiting among HIV/AIDS patients are very diverse, awareness of this phenomenon is useful in diagnosing and managing the problem.

## Background

Causes of nausea and vomiting among patients with human immunodeficiency virus (HIV) infection and/or acquired immune deficiency syndrome (AIDS) are diverse; gastrointestinal, neurological, endocrine causes to name a few [1]. Nephrotoxicity and proximal renal tubular acidosis (RTA) known as Fanconi syndrome are known and established side effect of tenofovir (TDF), a nucleotide analog [2–5]. One study reported that the incidence rate of moderate or severe renal insufficiency caused by tenofovir was 29.2 and 2.2 cases per 1000 person-year respectively [6]. Fanconi syndrome caused by tenofovir is rare and exact incidence rate is not known. Fanconi syndrome caused by tenofovir is usually associated with other manifestations such as worsening of glomerular filtration rate (GFR), proteinuria, hypophosphataemia, and glycosuria [4, 5].

RTA can be differentiated into either distal or proximal, depending on the site of tubules affected. Both have normal serum anion gap, but distal RTA is characterized by positive urine anion gap ([Na^+^ + K^+^]_u_ – [Cl^−^]_u_), whereas proximal RTA has negative urine anion gap [7]. Major causes of distal RTA in adults is autoimmune diseases such as Sjögren’s syndrome [7]. Medications such as ibuprofen are also known to cause distal RTA [8], but distal RTA caused by antiretroviral medications has never been reported to the best of our knowledge. We here present such a rare case of distal RTA most likely caused by tenofovir.

## Case presentation

A 20-year-old man with HIV infection visited us with chief complaint of nausea and vomiting. He had been followed at our Infectious Diseases clinic. He had no past medical history otherwise, including opportunistic diseases. Two months after his initial visit, antiretroviral therapy (ART) was started with Truvada ® (tenofovir and emtricitabine), and raltegravir. His baseline CD4+ T lymphocytes count before initiation of ART was 456/μL and RT-PCR for HIV-1 was 2.8 x 10^3^ copies/mL. Other routine blood and urine tests were all normal. He stated that he had been adherent to ART. Six weeks after initiation of ART, he developed nausea and vomiting. He visited our Infectious Diseases clinic on the following day. He denied fever, headache, seizure, abdominal pain, or diarrhea. He also denied use of medications other than we prescribed, supplements, alcohol or any illicit drugs.

On physical examination, he appeared anxious. Blood pressure was 116/70 mmHg, pulse rate 95/min, respiratory rate 18/min, and body temperature 36.9 °C. Rest of physical examination including thorough neurological examination was normal. On blood tests, complete blood counts (CBC), electrolytes, liver and kidney function tests were all unremarkable (Table 1). Arterial blood gas showed pH 7.327, pCO2 38.3 mmHg, bicarbonate 19.3 mmol/L, and anion gap 13.9 mmol/L. Spot urianalysis showed urine pH 5.5, and urine sodium 160 mmol/L, potassium 52 mmol/L, chloride 48 mmol/L, with urine anion gap ([Na^+^ + K^+^]_u_ – [Cl^−^]_u_) of 164 mmol/L. Urine phosphorus was 138.4 mg/dL, urine calcium was 31.9 mg/dL, and urine β2-microglobulin was 1268 μg/L (normal range 0-289). Urine was also positive for protein (1+) and ketone (3+). Urine specific gravity was 1.032 with approximate urine osmolality of 1000 mOsm/kg [9]. Calculated urine osmolality (2 x [Na + K]) + [urea nitrogen in mg/dL]/2.8 + [glucose in mg/dL]/18) was 939 mOsm/kg [10], with approximate urine osmolal gap of 61 mOsm/kg.

**Table 1:** Laboratory Data

Variables	Reference Ranges, Adults	On the day of the visit	Next day
While-cell count (per mm^3^)	4000–8500	5100	
Hemoglobin (g/dL)	13.6–17	14.7	
Platelet (per mm^3^)	130,000–300,000	209,000	
Sodium (mmol/L)	137–146	136	
Potassium (mmol/L)	3.5–4.7	4.1	
Chloride (mmol/L)	99–109	103	
Calcium (mg/dL)	8.8–10.1	9.2	
Phosphorus (mg/dL)	2.4–4.5	4.1	
Albumin (g/dL)	4.1–5	5.2	
Aspartate aminotransferase (AST) (U/L)	13–31	17	
Alanine aminotransferase (ALT) (U/L)	8–34	15	
Total bilirubin (mg/dL)	0.3–1	1.2	
Blood Urea Nitrogen (BUN) (mg/dL)	9–22	19.3	
Creatinine (mg/dL)	0.5–1.3	0.59	
Blood gas		(arterial)	(venous)
pH	7.38–7.46	7.327	7.334
Partial pressure of carbon dioxide (PaCO_2_) (mmHg)	32–46	38.3	43.1
Bicarbonate (HCO_3_^−^) (mmol/L)	21–29	19.5	22.3
Anion-gap (mmol/L)	10–20	13.9	12.3

Non-anion-gap metabolic acidosis was considered to be the cause of his current symptoms, and medications were suspected as the causative agent. Intravenous hydration and antiemetic was provided and all antiretroviral agents were discontinued at once. On the following day, his symptoms disappeared. Venous blood gas revealed normalized bicarbonate (Table 1). Five days after this event, the patient re-started ART with Epzicom ® (abacavir and lamivudine), and ralteglavir. He remains on the latest regimen without further events. Four months after re-starting ART, his CD4+ T lymphocytes count increased to 648/μL with undetectable HIV-1RNA.

Tenofovir is known to cause drug induced Fanconi syndrome, but is usually associated with other manifestations, particularly worsening of glomerular filtration rate. However, our patient lacked these symptoms, and urine anion gap was positive, making proximal renal tubular acidosis such as Fanconi syndrome less likely.

In our case, acute onset nausea and vomiting *without* diarrhea, with non-anion-gap metabolic acidosis plus positive urine anion-gap, makes distal renal tubular acidosis most likely. The reason for lack of potassium abnormality in the patient remains unknown, but acute onset of symptoms and prompt treatment might have prevented significant potassium wasting, which is characteristic of most distal RTA. We consider this case is of distal RTA of unclassified type, with prompt improvement after cessation of causative agent; i.e. tenofovir. Since discontinuation of tenofovir was effective in prompt disappearance of symptoms and acidosis, and re-institution of lamivudine (analogous to emtricitabine. They are very similar both in structure and pharmacological properties) and raltegravir did not cause the recurrence of the symptoms, we considered tenofovir as the most likely cause of RTA in this patient. We obtained venous blood gas to ascertain the correction of acidosis since recent meta-analysis found that venous gas is comparable to arterial blood gas in evaluating pH [11].

This is the first case ever reported on such an occurrence as far as we know of. The exact mechanism of renal toxicity caused by tenofovir is unknown, but it is considered to be the result of proximal tubular toxicity [12]. Pathogenesis of distal RTA by tenofovir, as in our case, remains unknown. Diminished hydrogen adenosine phosphatase (H-ATPase) activity is the most common cause of distal RTA [13], and it might have occurred with use of tenofovir. Although not reported so far, other antiretroviral agents, particularly nucleoside/nucleotide analogs also might cause similar side effect. Further studies might reveal pathogenesis of this phenomenon, as well as the true incidence and significance of this side effect. In addition, use of newer and apparently safer agent, tenofovir alafenamide (TAF) may prevent this to occur [14].

## Conclusions

We here present a rare case of distal RTA without renal impairment most likely caused by tenofovir. Since causes of nausea and vomiting among HIV/AIDS patients are very diverse, awareness of this phenomenon is useful in diagnosing and managing the problem.

## Abbreviations

AIDS: Acquired immune deficiency syndrome
ART: Antiretroviral therapy
CBC: Complete blood counts
GFR: Glomerular filtration rate
H-ATPase: Hydrogen adenosine phosphatase
HIV: Human immunodeficiency virus
RNA: Ribonucleic acid
RTA: Renal tubular acidosis
TAF: Tenofovir alafenamide
TDF: Tenofovir

## References

[1] Fauci AS, Lane HC, Kasper DL (2015). Human Immunodeficiency Virus Disease: AIDS and Related Disorders. Harrison’s Principles of Internal Medicine.

[2] Karras A, Lafaurie M, Furco A, Bourgarit A, Droz D, Sereni D (2003). Tenofovir-related nephrotoxicity in human immunodeficiency virus-infected patients: three cases of renal failure, Fanconi syndrome, and nephrogenic diabetes insipidus. Clin Infect Dis.

[3] Atta MG, Stokes MB (2013). ASN Clinical Pathological Conference. CJASN.

[4] Hall AM, Bass P, Unwin RJ (2014). Drug-induced renal Fanconi syndrome. QJM.

[5] Luni FK, Khan AR, Prashar R, Vetteth S, Duggan JM (2016). Fanconi Syndrome and Antiretrovirals: It Is Never Too Late. Am J Ther.

[6] Quesada PR, Esteban LL, García JR, Sánchez RV, García TM, Alonso-Vega GG (2015). Incidence and risk factors for tenofovir-associated renal toxicity in HIV-infected patients. Int J Clin Pharm.

[7] DuBose TD, Kasper DL (2015). Acidosis and Alkalosis. Harrison’s Principles of Internal Medicine.

[8] Salter MD (2013). Ibuprofen-Induced Hypokalemia and Distal Renal Tubular Acidosis: A Patient’s Perceptions of Over-the-Counter Medications and Their Adverse Effects. Case Rep Crit Care.

[9] Leech S, Penney MD (1987). Correlation of specific gravity and osmolality of urine in neonates and adults. Arch Dis Child.

[10] Dyck RF, Asthana S, Kalra J, West ML, Massey KL (1990). A modification of the urine osmolal gap: an improved method for estimating urine ammonium. Am J Nephrol.

[11] Byrne AL, Bennett M, Chatterji R, Symons R, Pace NL, Thomas PS (2014). Peripheral venous and arterial blood gas analysis in adults: are they comparable? A systematic review and meta-analysis. Respirology.

[12] Lucey JM, Hsu P, Ziegler JB. Tenofovir-related Fanconi’s syndrome and osteomalacia in a teenager with HIV. BMJ Case Reports. 2013;bcr2013008674. https://www.ncbi.nlm.nih.gov/pmc/articles/PMC3736206/. 10.1136/bcr-2013-008674 PMC3736206 23843401

[13] Emmett M and Kelepouris E. Overview and pathophysiology of renal tubular acidosis and the effect on potassium balance. UpToDate. [cited 2016 Sep 23]. last updated: Jun 30, 2015.

[14] De Clercq E. Tenofovir alafenamide (TAF) as the successor of tenofovir disoproxil fumarate (TDF). Biochemical Pharmacology [Internet]. [cited 2016 May 18]; Available from: http://www.sciencedirect.com/science/article/pii/S0006295216300697. Accessed 30 June 2015. 10.1016/j.bcp.2016.04.015 27133890

